# Editorial: Cannabis Genomics, Breeding and Production

**DOI:** 10.3389/fpls.2020.591445

**Published:** 2020-10-30

**Authors:** Rachel Backer, Giuseppe Mandolino, Olivia Wilkins, Mahmoud A. ElSohly, Donald L. Smith

**Affiliations:** ^1^Department of Plant Science, McGill University, Montreal, QC, Canada; ^2^Research Center for Industrial Cultures, Council for Agricultural Research and Analysis of Agricultural Economics, Bologna, Italy; ^3^Department of Biological Sciences, University of Manitoba, Winnipeg, MB, Canada; ^4^School of Pharmacy, University of Mississippi, Oxford, MS, United States

**Keywords:** cannabinoids, cultivar development, photobiology, fertilizer application, plant pathology, plant growth-promoting rhizobacteria, polyploid, flower induction

## Introduction

*Cannabis sativa* was illegal during most of the 20th century, but has recently been decriminalized or even legalized in some jurisdictions. During the same period, scientific tools were developed, giving us unprecedented insights into how plants grow, evolve, interact with their environment, and synthesize metabolites. However, because cannabis was largely illegal as these advances were made, this plant has been woefully understudied, and continues to hold many mysteries. To move forward, and bring the benefits of cannabis to the forefront, the legal landscape must be streamlined to allow for efficient scientific investigation.

The legal classification of cannabis and hemp in the United States (Mead) and around the world is rapidly evolving which means there are ever-changing obstacles for producers and researchers alike. For example, in the US, there is confusion as to whether cannabis state laws are superseded by federal law, a variety of factors that determine the extent of enforcement related to state-authorized cannabis activities, and questions surrounding the legality and approval process for CBD-based products. Also in Europe the relations between EU regulations and controls, and the attitude of national legislations toward cannabis is not without contradictions. In addition, cannabis literature is surrounded by relics of black-market terminology mixed with current pharmaceutical influences that make for an unusual landscape (Russo). For example, referring to cannabis “strains” is a misnomer and they would more appropriately be termed “chemovars.” In addition, the notion that cannabinoid biosynthesis in yeast can replace cultivation of whole plants may be an oversimplification that relies on the assumption that the benefits of cannabis-based medicines come from single compounds. These legal and conceptual frameworks must be addressed to streamline the advance of research and adoption of cannabis-based medicines.

To date, much research on cannabis has focused on distinguishing between marijuana (drug-type cannabis) and hemp (fiber/seed-type cannabis) (Gilmore et al., 2003; Datwyler and Weiblen, 2006; Howard et al., 2009; Rotherham and Harbison, 2011; Sutipatanasomboon and Panvisavas, 2011; Sawler et al., 2015; Dufresnes et al., 2017), quantifying cannabinoids accumulation in plant tissues (Mahlberg and Kim, 2004; Pacifico et al., 2008; Muntendam et al., 2012; Happyana, 2014; Happyana and Kayser, 2016) and elucidating cannabinoid biosynthesis (Flores-Sanchez and Verpoorte, 2008; Marks et al., 2009; Flores-Sanchez et al., 2010). This reflects the fact that drug-type cannabis was illegal and needed to be rapidly distinguished from hemp in the context of law enforcement. However, there are a few examples of studies that examined how to elicit cannabinoid or terpenoid biosynthesis (Lydon et al., 1987; Mansouri et al., 2009a,b, 2011, 2013, 2016; Mansouri and Asrar, 2012; Mansouri and Rohani, 2014; Mansouri and Salari, 2014), how fertilization affects cannabis and hemp yields (Finnan and Burke, 2013a,b; Aubin et al., 2015; Campiglia et al., 2017; Caplan et al., 2017), classification of cannabis varieties based on chemotype (Choi et al., 2004a,b; Fischedick et al., 2010; Hazekamp and Fischedick, 2012; Hazekamp et al., 2016), and large-scale genome sequencing efforts (Van Bakel et al., 2011; McKernan et al., 2020). The research in this volume extends on these topics to improve our understanding of applications of novel production, breeding, and analytic tools can improve cannabis and hemp cultivation.

## Production Factors That Influence Cannabis Yield and Quality

A wide variety of specialty cannabis fertilizers are used but efficacy of these products and techniques remain largely scientifically unproven. Questions considered in this volume include: how do different genotypes of cannabis respond to the level of K fertilization? Do nutritional supplements such as humic acid supplementation or inorganic N, P or K affect plant cannabinoid profile? To address the first question, two cannabis genotypes were fertilized with five levels of K (Saloner et al.) (ranging from 15 to 240 ppm K). Growth responses showed that response to K level varied between genotypes but that 15 ppm K was too low for both genotypes leading to growth reduction. However, this effect was associated with contrasting mechanisms in the two genotypes. In contrast, 240 ppm K was toxic to one genotype but stimulated root and shoot development in the other. The higher K tolerance of the second genotype appeared to be associated with higher levels of K transport from root to shoot. To address the second question, the effects of humic acids and inorganic N, P and K on cannabinoid profiles (Bernstein et al.) throughout the plant were studied using three enhanced nutrition treatments compared to a commercial control treatment. The results of this study confirm that nutrition supplementation in cannabis can contribute to standardize cannabinoid biosynthesis.

Cannabis plants are susceptible to a variety of pathogens (fungal and bacterial) and insect pests that contribute significantly to yield losses. This is a particularly difficult challenge to address due to the nature of hydroponic growing systems where natural predators do not exist, and the use of chemical control strategies is undesirable because of the residues left on flowers. The first step toward developing better pathogen control strategies is to gain a clear picture of the pathogens present in cannabis cultivation. One paper in this volume took stock of pathogens and molds that affect cannabis production (Punja et al.) in indoor hydroponic systems and in field-grown plants and investigated how pathogens are introduced into, spread within, and become established in indoor cultivation systems.

To understand how cannabis production can be improved, we first need to understand if producers are achieving optimum crop yields. This meta-analysis (Backer et al.) showed that current statistics reported by cannabis producers appear to be projections based on facility size—these yields appear to be substantially higher than yields obtained in scientific studies which begs the question of whether these yields are being obtained in industry. If they are, scientists need to collaborate with industry to better understand state-of-the art cultivation methods. If these projected yields are not being obtained, scientists can help determine how to achieve them. To date, the literature suggests that biomass and cannabinoid yields vary considerably depending on variety, plant density, light intensity and fertilization while the meta-analysis also revealed pot size, light type, and duration of the flowering period as predictors of yield and THC accumulation. Another article in this topic considers the role of photobiology in cannabis cultivation (Bilodeau et al.) and highlights the role of light wavelength, intensity and photoperiod on plant photosynthesis and photomorphogenesis through plant photoreceptors. The authors suggest that lighting practices can be improved for cannabis production, for example, by altering the spectra of LED lights to stimulate photoreceptors to maximize cannabis yield and quality while reducing operation costs. Novel inputs can also be developed to improve cannabis yields, such as the application of plant growth-promoting rhizobacteria (PGPR) (Lyu et al.) which have contributed to yield increases in other cultivated crops. For example, members of *Bacillus* or *Pseudomonas* may improve cannabis and hemp yield and/or quality via direct growth stimulation, improved nutrient acquisition and/or biological control of pathogens. Finally, propagation of vigorous, uniform plants remains a challenge for the cannabis industry (Chandra et al.) because this crop is dioecious and relies on cross-fertilization for seed production. This article provides a summary of propagation strategies for indoor and outdoor cultivation including vegetative and micropropagation methods.

## Breeding Considerations

Another challenge facing the cannabis industry is the need to develop new cultivars with desirable cannabinoid profiles, high productivity and pest resistance, and overall vigor. While polyploidization has been used successfully in hemp breeding, it had not been attempted in cannabis. This volume contains the first recorded application of tetraploid drug-type cannabis lines (Parsons et al.). Fan and sugar leaf sizes were increased on tetraploid clones but these leaves had lower stomata and trichome densities, respectively, compared to diploid clones. While tetraploid clones had higher CBD concentrations in buds and significantly different terpene profiles compared to diploid clones, dry bud yield and THC content were similar. These findings provide a strong footing and a new tool for cannabis breeding programs.

In the case of hemp, yield and quality are largely determined by the cultivar, but environmental factors such as temperature and photoperiod also have strong influences on these parameters. Molecular breeding strategies *via* a candidate gene approach for the development of cultivars adapted to specific geographical regions (Salentijn et al.) can make use of current phenotypic and genetic data. For example, it appears that several key genes control traits such as flowering behavior and that natural genetic variation may allow for development of varieties with specific flowering times.

## Biology of Cannabis

Cannabis is considered a facultative short-day plant: growers use long photoperiods during propagation and vegetative growth phases and induce flowering using shorter photoperiods. However, new research showed that induction of flowering was age-dependent (Spitzer-Rimon et al.) and likely controlled by internal signals rather than photoperiod for two medical cannabis cultivars. They also demonstrated that there is natural variation in cannabis architecture and inflorescence termination and suggest that a short photoperiod results in intense inflorescence branching but is not necessarily responsible for floral initiation. Together these findings suggest that cannabis may be considered under some circumstances as a day-neutral plant and provide a deeper understanding of cannabis inflorescence development.

A major challenge in breeding new cannabis and hemp cultivars lies in the poor understanding of the phylogeographic structure and domestication of cannabis. Zhang et al. described three haplogroups, from wild and domesticated populations or cultivars, which were associated with distinct high-middle-low latitudinal gradient distribution patterns and consistent with the existence of three cannabis subspecies (*C. sativa* subsp. *ruderalis, sativa*, and *indica*). Day-length was found to be the most important factor influencing population structure. The paper also suggests that there are multiregional origins for domesticated cannabis and that cannabis probably originated in a low-latitude region.

## Chemical Analysis of Cannabinoids and Terpenoids

There is strong potential for the use of metabolomics, or cannabinomics (Aliferis and Bernard-Perron), to be used in cannabis taxonomy, for example to develop a chemovar classification system. Other possible applications include characterization and discovery of new cannabis-based bioactive molecules for medical use, for food, and for optimizing cannabis cultivation. For example, in this topic, researchers characterized the plasticity of alkyl cannabinoid composition across plant tissues and developmental stages and found a range of di-/tri- cyclic and C_3_-/C_5_-alkyl cannabinoids in plants. The composition of cannabinoids varied between plants, however, the chemotype at the vegetative and flowering growth stages were predictive of the chemotype at maturity. The results suggest that there is a low level of plasticity in cannabinoid composition (Welling et al.). Furthermore, liquid chromatography-high-resolution mass spectrometry analysis of ten commercially available organic hemp seed oils revealed the presence of THC, CBD, and 30 other cannabinoids; these were detected for the first time in hemp seed oil (Citti et al.) using an untargeted metabolomic approach. This highlights that we still have much to learn about cannabis chemical composition as we apply new analytic tools to this ancient crop; this knowledge will allow us to improve the pharmaceutical value of medicinal cannabis and the health properties of hemp-based foods.

## Author Contributions

RB and DS developed the overall concept. RB wrote the first complete draft. GM, OW, and ME provided input and comments to the draft. DS provided input and feedback on subsequent drafts. All authors contributed to the article and approved the submitted version.

## Conflict of Interest

The authors declare that the research was conducted in the absence of any commercial or financial relationships that could be construed as a potential conflict of interest.
